# Cellular Automata Modeling of Ostwald Ripening and Rayleigh Instability

**DOI:** 10.3390/ma11101936

**Published:** 2018-10-11

**Authors:** Fengbo Han

**Affiliations:** Max-Planck-Institut für Eisenforschung GmbH, Max-Planck-Straße 1, D-40237 Düsseldorf, Germany; hanfengbo@163.com

**Keywords:** Ostwald ripening, Rayleigh instability, cellular automata, curvature-driven

## Abstract

A cellular automata (CA) approach to modeling both Ostwald ripening and Rayleigh instability was developed. Curvature-driven phase interface migration was implemented to CA model, and novel CA rules were introduced to ensure the conservation of phase volume fraction of nearly equilibrium two-phase system. For transient Ostwald ripening, it is shown that the temporal growth exponent m is evolving with time and non-integer temporal exponents between 2 and 3 are predicted. The varying temporal growth exponent m is related to the particle size distributions (PSDs) evolution. With an initial wide PSD, it becomes narrowed toward steady state. With an initial narrow PSD, it becomes widened at first and then narrowed toward steady state. For Rayleigh instability, two cases (one with sinusoidal perturbation on the surface of the long cylinder, and the other with grain boundaries in the interior of the long cylinder) were simulated, and the breakup of the long cylinder was shown for both cases. In the end, a system containing long cylinders with interior grain boundaries was simulated, which demonstrated the integration of Rayleigh instability and Ostwald ripening relating to the spheroidization of the lamellar structure.

## 1. Introduction

Ostwald ripening and Rayleigh instability are two distinct processes, but with the same driving force coming from the reduction in the interface/surface area. The former is an observed phenomenon for a system with second-phase particles of various sizes dispersed in a matrix, in which larger particles grow at the expense of the smaller ones. The latter is a phenomenon of the breakup of rod-like (high-aspect-ratio) structures due to the progressive growth of axial perturbations, leading to these rod-like structures breaking up into an array of equiaxed structures [1]. Modern engineering materials often contain high-aspect-ratio features (e.g., titanium alloys [2,3] and TiAl alloys [4,5] with lamellar structures, Al–Si Alloys [6] with eutectic microstructure, fiber reinforced composites [7]). When applied at elevated temperatures, the high-aspect-ratio structures in these materials undergo transformation which could ultimately lead to their breakup. Microstructural changes of materials at high temperature may deteriorate the mechanical properties, so the instabilities of the high-aspect-ratio structures in these materials pose a threat to their high-temperature applicability. When subjected to thermomechanical processing, the high-aspect-ratio structures in these materials are much easier to dissociate. After dissociation, the second phase particles disperse in the matrix and still undergo transformation which leads to the growth of large particles and shrinkage of small particles. In this way, the microstructures of materials can be tailored as well as the properties. Both Rayleigh instability and Ostwald ripening play important roles in microstructure control and performance evaluation of materials with high-aspect-ratio features. Better understanding and modeling of the two processes is of great fundamental and practical interest.

A classical theory of Ostwald ripening is known as LSW theory, which was done by Lifshitz and Slyozov [8] and by Wagner [9]. This theory is limited to the steady state of Ostwald ripening for the extreme case with vanishingly low particle fraction, and predicts the growth law, ${\langle R\left(t\right)\rangle }^{m}-{\langle R\left(t_{0}\right)\rangle }^{m}=Kt$, with the exponent *m* = 3, where 〈R〉 is the average particle radius, *t* is the time and K is the coarsening rate constant. The most recent model was developed by Ardell and Ozolins [10] and later elaborated by Ardell [11,12,13,14]. They assume that the coarsening process is trans-interface-diffusion-controlled (TIDC), instead of the classical theory with the assumption of matrix diffusion controlled. The TIDC theory predicts the exponent m of the growth law in the range of 2 and 3 either by invoking a particle size-dependent interface width [11,12] or by invoking concentration-dependent diffusion coefficient [14]. Apart from analytical models, numerical models of Ostwald ripening have been reported, such as phase field model [15,16,17,18], Monte Carlo model [19,20,21,22], and cellular automata (CA) model [23]. One of the advantages of these numerical models is that they can consider more realistic conditions, such as the initial PSD and particle morphology.

Lord Rayleigh [1] investigated the classic instability that causes the cylindrical fluid jets to break up into spherical droplets, which is responsible for the common phenomenon of pinch-off of thin water jets emerging from kitchen taps. Nichols and Mullins [24,25] studied the Rayleigh instability in solid, and they formulized the morphological changes of solid cylinders taking into account mass transport by surface diffusion and volume diffusion. For an infinite cylinder of radius R0, subjected to a sinusoidal radial perturbation of the form $r=R_{0}+\delta \sin\left(2\pi x/\lambda \right)$, only when the wavelength λ > 2π*R*_0_, the amplitude of the perturbation can increase spontaneously, and finally leading to the breakup of the cylinder. Recently, the phase field model has been used to study the Rayleigh instabilities in the solid state [26,27,28,29,30,31]. Joshi et al. [26,27] formulated phase field models for studying the evolution of cylindrical pore in both homogeneous solid and polycrystalline membrane. Chakrabarti et al. [28] used phase field to study the grain boundary driven Rayleigh instability in the multilayer nanocrystalline thin film. Wang and Nestler [29] proposed a generalized stability criterion for nanowires and conducted phase field simulations to confirm it. Amos et al. [30,31] conducted a phase field analysis of shape-instabilities in metallic systems with 2-dimensional plate-like structures and finite 3-dimensional rods.

Although only limited attention has been given to modeling both Ostwald ripening and Rayleigh instability using the CA method, it still shows some advantages. CA describes the evolution of complex systems by applying local transformation rules to the cells of a lattice [32]. It is very flexible to make appropriate rules based on the physical mechanism of the actual system and easy to calibrate to real time and length scale. The governing equations in CA models are physically intuitive and easily implemented. The CA model can describe the more realistic microstructural morphology of a system when compared to the mean-field model which is based on the simple artificial geometrical assumption (e.g., circular grain or particle). The computational efficiency of the CA model is more attractive when compared to the phase field model. It is convenient to couple CA models with finite element method (FEM) [33,34,35,36], which makes it suitable for engineering applications. What is more, CA models have been successfully applied to simulate grain growth based on the curvature-driven mechanism [37,38,39,40,41,42,43,44,45], and it is much promising to extend the CA method to model Ostwald ripening and Rayleigh instability, which are also curvature-driven phenomena. 

In this paper, we present a novel cellular automata approach to modeling both Ostwald ripening and Rayleigh instability based on the curvature-driven mechanism. Physical formulas to describe curvature-driven phase interface migration for a two-phase system of equilibrium composition and volume fraction were derived. Novel CA rules were made to ensure the conservation of the phase volume fraction of nearly equilibrium two-phase system. Typical cases were studied to confirm the ability of the developed model to simulate both Ostwald ripening and Rayleigh instability. The kinetics and the PSD evolutions during Ostwald ripening were discussed. The successive occurrence of Rayleigh instability and Ostwald ripening in a system with long cylinders was captured by the model.

## 2. Modeling

### 2.1. Curvature-Driven Phase Interface Migration

For a two-phase (*α* + *γ*) system, when transformation between *α* phase and *γ* phase occurs, the *α*/*γ* interface velocity can be described by [46]

(1) $$v_{\alpha \gamma }=M^{\alpha \gamma }\Delta G$$

where $M^{\alpha \gamma }$ is the interface mobility and ΔG is the chemical driving force. The mobility is assumed to follow the relationship [47]

(2) $$M^{\alpha \gamma }=M_{0}^{\alpha \gamma }\exp\left(-Q_{\alpha \gamma }/RT\right)$$

where $M_{0}^{\alpha \gamma }$ is the pre-exponential factor of the interface mobility, $Q_{\alpha \gamma }$ is the activation energy for boundary migration, *R* is the universal gas constant (8.314 J mol^−1^ K^−1^), and *T* is the absolute temperature.

The chemical driving force, Δ*G*, is assumed to be proportional to the content difference on the moving interface with respect to the equilibrium content by a first approximation [48,49]. For *γ* phase grows into *α* phase (*α*→*γ*), the driving force for the interface can be written as [48,49]

(3) $$\Delta G=\chi_{\left(T\right)}\left(x^{\gamma /\alpha }-x^{\gamma }\right)$$

where $\chi_{\left(T\right)}$ is a proportionality factor, and *x^γ^*^/*α*^ and *x^γ^* are the solute concentration at the moving *γ*/*α* interface and the equilibrium concentration, respectively.

Considering a system with disperse second phase (*α* phase) particles statistically distributed in a matrix (*γ* phase) and possessing certain solubility in it, which will be thermodynamically unstable due to a large interface area [50]. In approaching equilibrium, the total interface area decrease accompanied by particle coarsening, and the solubility depends on particle radii, which is described by the well-known Gibbs–Thomson equation

(4) $$x_{r}=x_{0}\exp\left(\frac{2\gamma_{\alpha \gamma }V_{m}}{RT}\cdot \frac{1}{r}\right)\approx x_{0}\left(1+\frac{2\gamma_{\alpha \gamma }V_{m}}{RT}\cdot \frac{1}{r}\right)$$

where *x*_0_ is the solute concentration at a plane interface in the matrix in equilibrium with particle of infinite radius, *x_r_* is the solubility at the surface of a spherical particle with radius *r*, *γ_αγ_* is the interfacial energy of the matrix-second phase boundary, and *V_m_* is the mean molar volume of the particle.

Equation (4) can be generalized to any phase interface with curvature *κ*,

(5) $$x_{\kappa }=x_{0}\left(1+\frac{\gamma_{\alpha \gamma }V_{m}}{RT}\cdot \kappa \right)$$

For the aforementioned nearly equilibrium two-phase system, *x_γ_*_/*α*_ and *x_γ_* in Equation (3) are equivalent to *x_κ_* and *x*_0_ in Equation (5), and the driving force Δ*G* can be rewritten as

(6) $$\Delta G=\chi_{\left(T\right)}\cdot x_{0}\frac{\gamma_{\alpha \gamma }V_{m}}{RT}\cdot \kappa$$

then the phase interface velocity can be rewritten as

(7) $$v_{\alpha \gamma }=\xi \cdot M^{\alpha \gamma }\gamma_{\alpha \gamma }\kappa$$

where $\xi =\chi_{\left(T\right)}x_{0}V_{m}/RT$, which will be a constant at a given temperature.

Apparently, the phase interface velocity during the transformation of *α*→*γ* is described in a curvature-driven form. This provides the possibility to simulate Ostwald ripening by just dealing with interface curvature, instead of dealing with solute concentration. In Section 2.3 additional CA rules will be introduced to realize the transformation of *γ*→*α*. By integration of the curvature-driven phase interface migration mechanism and appropriate CA rules, the microstructure evolution of two-phase system during Ostwald ripening can be simulated. It should be noted that although the equations are derived for the case of Ostwald ripening, it is also applicable for the case of Rayleigh instability, because the two-phase system of equilibrium concentration and volume fraction is also assumed for Rayleigh instability, and the driving forces for these two phenomena are the same.

### 2.2. Curvature-Driven Grain Boundary Migration

For the partial wetting two-phase system, the second phase (*α* phase) particles will impinge upon each other and form grain boundaries. These grain boundaries are more likely to be driven further away by the curvature, rather than remain in their local mechanical equilibrium [51,52]. For these curvature-driven grain boundary migrations, the velocity of a grain boundary segment can be expressed by

(8) $$v_{\alpha \alpha }=M^{\alpha \alpha }F$$

where *M_αα_* is the grain boundary mobility between grains of *α* phase, and *F* is the driving force originating from grain boundary curvature, which is expressed by

(9) $$F=\gamma_{\alpha \alpha }\kappa$$

where *γ_αα_* is the grain boundary energy.

As grain boundaries may have different misorientations, both the grain boundary mobility *M_αα_* and grain boundary energy *γ_αα_* are dependent on the boundary misorientation angle *θ*. The grain boundary mobility *M_αα_* is assumed to be as follows [53]

(10) $$M^{\alpha \alpha }\left(\theta \right)=M_{\mathrm{HAG}}\left(1-\exp\left(-5{\left(\frac{\theta }{\theta_{m}}\right)}^{4}\right)\right)$$

where *M*_HAG_ is the mobility of high-angle boundary with misorientation greater than *θ*_m_, which can be estimated as follows

(11) $$M_{\mathrm{HAG}}=\frac{D_{0}b^{2}}{k_{B}T}\exp\left(-\frac{Q_{b}}{RT}\right)$$

where *Q_b_* is the activation energy for grain boundary motion, *D*_0_ is the boundary self-diffusion coefficient, *b* is the magnitude of the Burgers vector, and *k*_B_ is the Boltzmann’s constant.

The misorientation dependent grain boundary energy *γ_αα_* can be calculated by the Read–Shockley equation [54,55]

(12) $$\gamma_{\alpha \alpha }=\gamma_{m}\left(\frac{\theta }{\theta_{m}}\right)\left(1-\ln\left(\frac{\theta }{\theta_{m}}\right)\right)$$

where *γ_m_* is the high-angle boundary energy.

As the model focuses on the curvature-driven interface motion mechanism, calculation of the interface curvature is one of the main tasks. A discrete disk template method is employed with the following equation [56,57]

(13) $$\kappa =\frac{3\pi }{c}\left(\frac{A}{A_{tot}}-\frac{1}{2}\right)$$

where *c* is the radius of the disk template, *A* is the area of the disk template outside the interface segment, and *A_tot_* is the area of an analytical disk with radius *c*.

### 2.3. The CA Model

In this work, the equations outlined above are implemented to a 2D CA model with the square cell. Each cell represents a material point and has several attributes (e.g., phase type and orientation). When a cell is located at the interface/boundary, it also has a transformation fraction variable f. The state variables for all lattice cells are updated in each time step, and a deterministic transformation rule used by Zheng et al. [58] is adopted to determine the changing state of each CA cell. For the cell with indices (*i*, *j*) that belong to the moving interface, the moving distance in a single time step, Δ*t*, is described as:

(14) $$l_{i,j}^{t}=\int_{t}^{t+\Delta t}vdt$$

where *v* is the interface velocity. The transformation fraction $f_{i,j}^{t}$ is then calculated by:

(15) $$f_{i,j}^{t}=l_{i,j}^{t}/L_{CA}$$

where *L_CA_* is the distance between two neighboring cells. The interface cell will switch into the new state when the transformation fraction reaches 1.0.

The aforementioned governing equations for the curvature-driven interface and grain boundary migration just realize the transformation of *α*→*γ* and *α*→*α*, respectively. To keep the conservation of phase volume fraction of the nearly equilibrium two-phase system, additional CA rules are needed to describe the transformation of *γ*→*α*. A two-step updating strategy is proposed in the present model. First, the states of *α* cells located at the *α*/*γ* interface and *α*/*α* boundaries are updated following the mechanism of curvature-driven interface and grain boundary migration, respectively; the number ($N_{cell}^{\alpha \to \gamma }$) of cells transform from *α* phase to *γ* phase is counted. Second, a certain number ($N_{cell}^{\gamma \to \alpha }$) of *γ* cells located at the *α*/*γ* interface are selected and forced to transform from *γ* phase to *α* phase. The selection of the *γ* cells obeys the principle that the local curvature will decrease after transformation. In the present work, a simplified approach is implemented. The *γ* cells located at the *α*/*γ* interface are randomly selected, and *γ* cells with more neighbor *α* cells have a higher probability to be selected than those that have less neighbor *α* cells. The key point here is to ensure that $N_{cell}^{\gamma \to \alpha }$ equals $N_{cell}^{\alpha \to \gamma }$, which guarantees the conservation of phase volume fraction. During the first step, the transformation of *α*→*γ* (which leads to the shrinkage of particles) mainly takes place at the *α*/*γ* interface of small-sized particles, since small-sized particles have large curvatures. During the second step, the transformation of *γ*→*α* (which leads to the growth of particles) mainly takes place at the *α*/*γ* interface of large-sized particles, since large-sized particles have a higher proportion of *α*/*γ* interface and interface *γ* cells located at the *α*/*γ* interface of large-sized particles have a higher probability to be selected during the mostly random selection process. The shrinkage of small-sized particles and the growth of the large-sized particles are the comprehensive results of the two steps. In brief, the shrinkage of small-sized particles is realized by the governing equations of curvature-driven interface migration, and the growth of large-sized particles is realized by a mostly random operation, and Ostwald ripening is realized by the integration of the physically-based curvature-driven mechanism and the novel CA rules.

The current CA model only deals with the state transformation of the interface cells, and if the transformation of *α*→*γ* occurs, then the transformation of *γ*→*α* is triggered instantaneously, without considering the diffusion distance that may take time. So it is suited to describe the interface-transfer limited case rather than the matrix diffusion limited case.

The key parameters used in the simulation are listed in Table 1. It should be noted that although real values of parameters for ferrite and austenite in steel are used, the present simulations are not corresponding to the real process for the alloy. The usage of real parameters shows that the current CA model can be calibrated to real process conveniently. All the grain boundaries between *α* phase particles are set as high angle grain boundaries.

## 3. Results and Discussion

### 3.1. Ostwald Ripening

**Figure 1:**
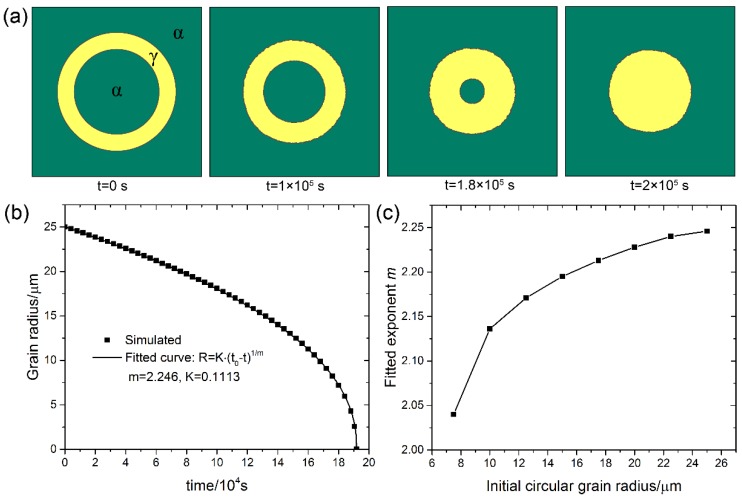
A typical two-phase system with circular *γ* phase embedded in the *α* matrix. (**a**) Microstructure evolution with time; (**b**) The inner grain radius vs. time; (**c**) Fitted exponent *m* of the function $R=K{\left(t_{0}-t\right)}^{1/m}$ for different initial radii of the inner grain. Simulation grid is 1000 × 1000 with the cell size of 0.1 μm.

A simple case for testing Ostwald ripening is a two-phase system with a circular phase embedded in the matrix, and Figure 1a shows the time evolution of such a system. The inner *α* grain shrinks toward its center with time, driven by the difference in curvatures of the outer interface and the inner interface (This actually exhibits the Gibbs–Thomson effect). The grain shape remains almost an ideal circle during the shrinkage, indicating that the present CA model successfully captures the curvature-driven mechanism. Figure 1b shows the inner grain radius vs. time, and it was fitted to a power-law, $R=K{\left(t_{0}-t\right)}^{1/m}$, where *R* is the grain radius, *t*_0_ is the time at which the grain disappears. The fitting gives *m* = 2.246. Fitted values of m for cases with different initial grain radii are presented in Figure 1c. The asymptotic trend of the exponent m toward the value of 2 with decreasing the initial grain radius is shown. The analytic model gives that *m* = 2 when the mass transport is interface-transfer limited and *m* = 3 when it is diffusion limited [59]. All the fitted values in Figure 1c are much closer to 2 than 3, indicating that the presented CA model successfully captures the interface-transfer mechanism.

**Figure 2:**
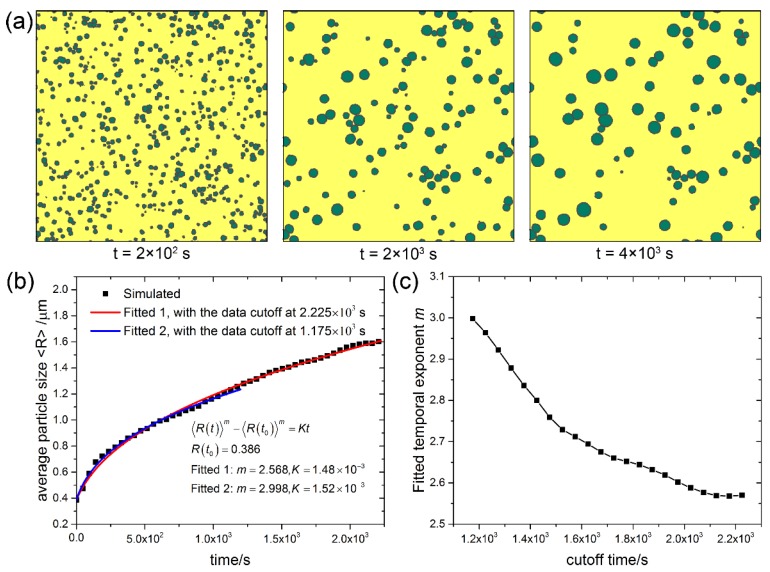
System with *f*_v_ = 10%. (**a**) Microstructure evolution; (**b**) Average particle size vs. time; (**c**) Fitted temporal exponents m of the power law ${\langle R\left(t\right)\rangle }^{m}-{\langle R\left(t_{0}\right)\rangle }^{m}=Kt$ at different cutoff times. Simulation grid is 1000 × 1000 with the cell size of 0.1 μm.

**Figure 3:**
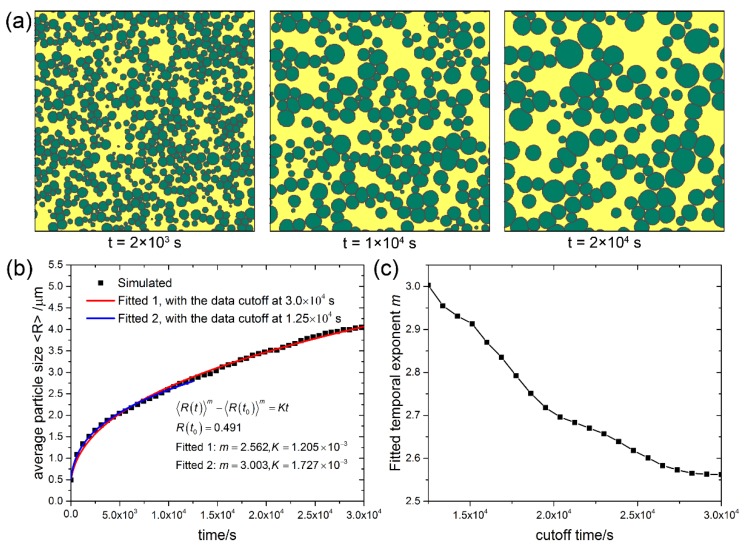
System with *f*_v_ = 50%. (**a**) Microstructure evolution; (**b**) Average particle size vs. time; (**c**) Fitted temporal exponents m of the power law ${\langle R\left(t\right)\rangle }^{m}-{\langle R\left(t_{0}\right)\rangle }^{m}=Kt$ at different cutoff times. Simulation grid is 1000 × 1000 with the cell size of 0.1 μm.

**Figure 4:**
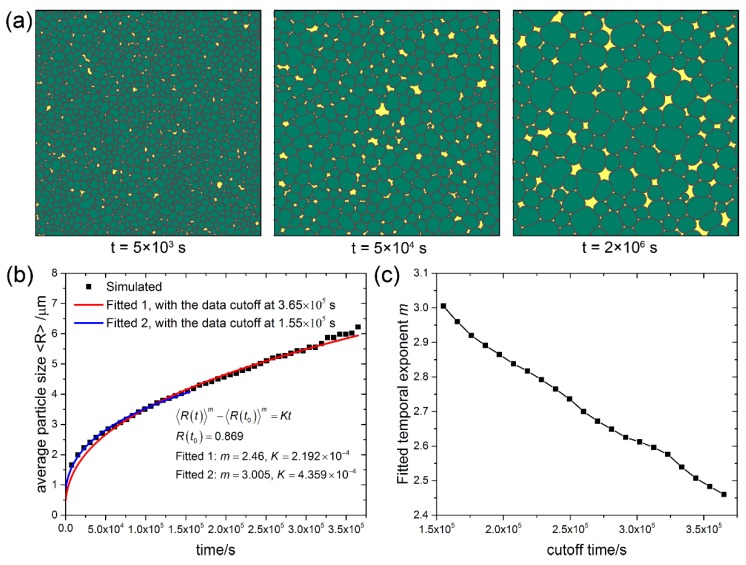
System with *f*_v_ = 90%. (**a**) Microstructure evolution; (**b**) Average particle size vs. time; (**c**) Fitted temporal exponents m of the power law ${\langle R\left(t\right)\rangle }^{m}-{\langle R\left(t_{0}\right)\rangle }^{m}=Kt$ at different cutoff times. Simulation grid is 1000 × 1000 with the cell size of 0.1 μm.

To investigate the ability of the current CA model in simulating Ostwald ripening for different volume fractions (*f*_v_) of second phase particles, systems with 10%, 50% and 90% volume fractions were simulated, and the microstructure evolution, time dependence of the average grain size, and the fitted temporal exponent of the three systems are shown in Figure 2, Figure 3 and Figure 4, respectively. It can be seen that at the volume fraction of 10% (Figure 2a), the grains of coarsening phase almost keep perfect circular morphology. As coarsening phase impingement is allowed in the present CA model, contact between grains of coarsening phase is observed in all cases. At the volume fraction of 50% (Figure 3a), although many grains of coarsening phase impinge with adjacent grains, most of the grains are still nearly circular. At the volume fraction of 90% (Figure 4a), shape accommodation is apparent for large growing grains, while small shrinking grains still keep nearly circular morphology. Some grain boundaries between large grains become flattened. Low volume fraction matrix phase stays at grain corners of the coarsening phase. Since each grain is represented by a unique orientation number in the CA model, there is no grain coalescence in all cases.

Most theories of Ostwald ripening are only applicable to systems approaching a scaling regime (steady state). In this regime, when scaled by the average radius, the particle size distribution (PSD) is time-independent or self-similar [18]. However, due to the limitation of computer power, when we do simulations only systems with limited size and a limited number of particles can be considered, and in this case, the steady state can hardly be reached. As it is true for simulations that the number of particles will decrease to hundreds or dozens before it reaches the steady state, which is more likely to approach the ultimate of Ostwald ripening. Even though the steady state can be reached, soon it will be broken when the number of particles decreases a lot. Considering the simulation scale in the current work and some phase field [16,17,18] studies on this issue, it is a high probability that the systems will not reach the steady state or a very short steady state occurs during the whole simulation process. So we do not try to detect whether and when the systems reach steady state, and the following discussions on Ostwald ripening are mainly limited to the transient stage.

Kinetics of Ostwald ripening in the transient stage show some differences from that in the steady state. The time dependence of the average particle size in systems with 10%, 50% and 90% volume fractions of the coarsening phase are shown in Figure 2b, Figure 3b and Figure 4b, respectively. The scatter data at different cutoff times are fitted to the power law ${\langle R\left(t\right)\rangle }^{m}-{\langle R\left(t_{0}\right)\rangle }^{m}=Kt$ with two variables m and *K*, and the non-linear fitted curves are plotted in solid lines. For the case of *f*_v_ = 10%, exponent *m* = 2.998 is fitted at the cutoff time of 1.175 × 103 s, while exponent *m* = 2.568 is fitted at the cutoff time of 2.225 × 10^3^ s. For cases of *f*_v_ = 50% and *f*_v_ = 90%, exponent *m* = 3 and 2 < *m* < 3 are also fitted at different cutoff times. More fitted exponent m at different cutoff times for the three cases are shown in Figure 2c, Figure 3c, and Figure 4c, respectively. It can be seen that the temporal exponent m varies in the range of 2 and 3, and shows a decreasing tendency with increasing the cutoff time. The current results show different temporal exponents for different cutoff times, which differs from the steady state theory of LSW theory that predicts the exponent *m* = 3. The varying temporal exponents can be ascribed to the varying PSDs (the evolution of PSDs will be investigated later on in the current work) in the transient stage of Ostwald ripening. It seems that the current results are more in favor of the TIDC theory that predicts varying temporal exponent. It was pointed out that the shape of PSDs and kinetic equations are intimately coupled by the TIDC theory [11]. This means that the TIDC theory has the ability to describe varying PSDs, and the best-fitted exponent values will be different for different shapes of PSDs. Ardell [11] fitted PSDs at varying aging times with the TIDC theory, and the best-fitted exponent values fluctuate in the range of 2 and 3, and there is no strictly monotonic relation. It should be noted that the temporal exponent fitted in the current work also does not show strictly monotonic decreasing property with time, as fluctuations can be observed in the trend of data shown in Figure 2c, Figure 3c, and Figure 4c. 

The decreasing trend of exponent m toward 2 can be explained that, as time increases, the number of particles in the systems decreases, and the kinetics of individual particle that shrinking or growing plays a more important role on the kinetics of the whole system. As shown in Figure 1c, the exponent of single particle shrinkage is close to 2. As long as the interface transfer limited case still remains, the exponent m will approach 2. However, Ardell and Ozolins [10] pointed out that, when particles grow to very large sizes, the coarsening behavior becomes matrix diffusion controlled. Because when the particles are very large, the concentration gradients in the matrix become so small that diffusion of solute in the matrix is actually slower than it is through the interface [14], and it will be matrix diffusion controlled instead of interface transfer controlled. So when the controlling mechanism changes to matrix diffusion, the current CA model is no longer applicable.

It should be noted that exponents m between 2 and 3 have been predicted theoretically for other reasons. Wagner [9] derived that *m* = 2 for extreme cases when Oswald ripening is interface reaction controlled. White and Fisher [60] and later White [61], considered the kinetics of coarsening in the transition region between interface control and diffusion control and predicted that the temporal exponent could vary in the range of 2 and 3. Sun [62] also considered the coarsening co-controlled by diffusion and a reversible interface reaction, and predicted a varying exponent between 2 and 3. Gusak et al. [63] analyzed Ostwald ripening with non-equilibrium vacancies, and showed that the exponent should be closed to 2 than to 3. However, the current CA model with deterministic rules is constructed based on the curvature-driven interface/boundary migration mechanism, and can be categorized as the interface-transfer limited case, which is totally different from theories that involve the transition between interface control and matrix diffusion control, or the case that diffusion is mediated by non-equilibrium vacancies.

When at the same fitted exponents (*m* = 3), the fitted coarsening rate constant *K* for 10%, 50% and 90% volume fractions are 1.52 × 10^−3^, 1.727 × 10^−3^ and 4.359 × 10^−4^, respectively. There is no obvious dependence of the coarsening rate constant *K* on the volume fraction of coarsening phase. This also differs from the LSW theory that predicts increased *K* for larger volume fractions. However, it is consistent with the TIDC theory that predicts independence of rate constants on volume fractions. It can be seen that the TIDC theory is able to describe the kinetics in the transient state of Ostwald ripening with varying PSDs.

**Figure 5:**
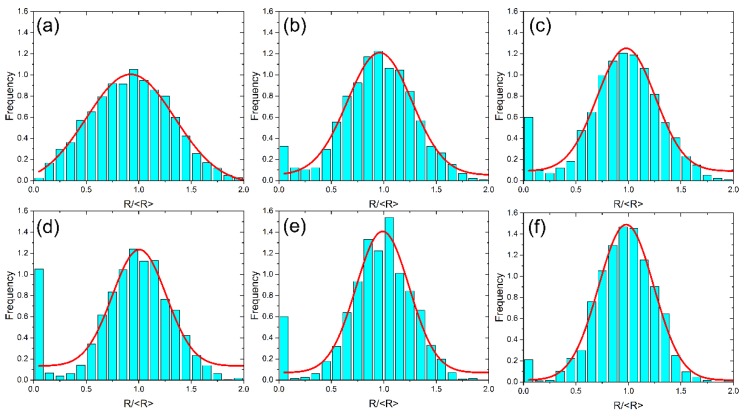
Particles size distributions at different times for the early stage in *f*_v_ = 90% coarsening phase system. (**a**) *t* = 0 s; (**b**) *t* = 2 × 10^2^ s; (**c**) *t* = 4 × 10^2^ s; (**d**) *t* = 1 × 10^3^ s; (**e**) *t* = 2 × 10^3^ s; (**f**) *t* = 5 × 10^3^ s.

As aforementioned, PSDs evolve during transient Ostwald ripening stage. Figure 5 shows the normalized PSDs at different times in *f*_v_ = 90% coarsening phase system. The heights of the bars represent the data of PSD while the solid curves are the Gaussian fits to these data. The initial PSD has a relatively wide shape, and it becomes narrower with time. It is noteworthy that the frequency of small particles increases at first, and then decreases, which can obviously be seen from the small particle size region of the histogram. The reason lies in that, small particles with radius lower than the critical radius (size with which the particle is momentarily neither growing nor shrinking) shrink with time before they completely disappear, the frequency of small particles will increase. After a number of small particles disappear, the frequency of small particles will decrease. On the contrary, the frequency for large particle size region (tail of the PSD) shows very small changes in the same period. Because large particles tend to grow by consuming small particles, they are much more stable than small particles. The decrease in the number of large particles will take a much longer time. Li et al. [18] studied the effect of the tail of PSD on the dynamics of transient Ostwald ripening, and they found that the initial tail of PSD controls the length of the transient coarsening stage. These all show the importance of the tail of PSD on the dynamics of transient Ostwald ripening.

**Figure 6:**
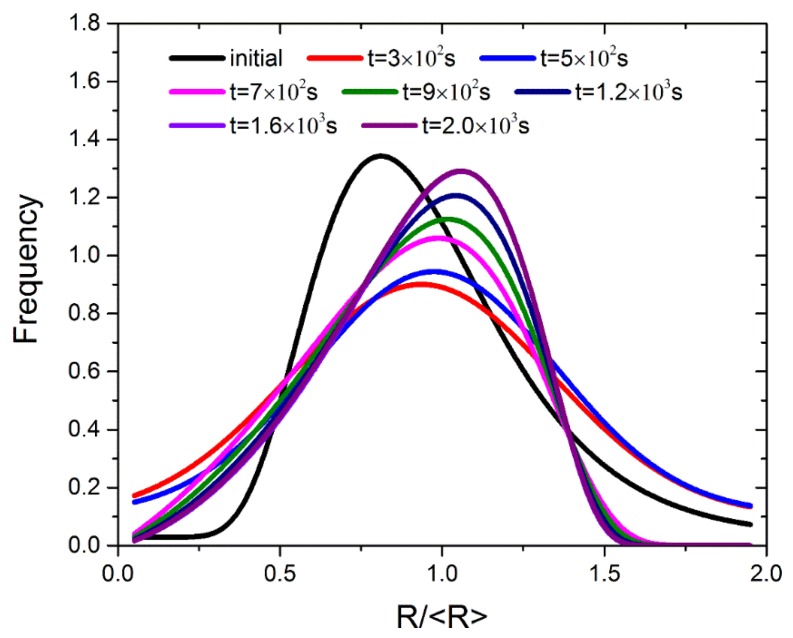
Time dependence of particle size distributions in *f*_v_ = 50% coarsening phase system.

Figure 6 shows the normalized PSDs at different times in *f*_v_ = 50% coarsening phase system. The PSDs are fitted to lognormal function, Gaussian function and the TIDC theory. Starting with a narrow shape of the PSD, it firstly becomes widened and then becomes narrowed again. With a narrow shape of initial PSD, differentiation will occur first, and the numbers of both smaller and larger particles will increase, making the shape of PSD widened. When a number of small particles disappear, the widened PSD will become narrowed again. After 1.6 × 10^3^ s, the PSDs almost overlap with each other, which means that it is near the steady state. In the phase field study of transient Ostwald ripening by Li et al. [18], this phenomenon was also observed when they conducted simulation with an initial PSD with the narrow shape.

**Figure 7:**
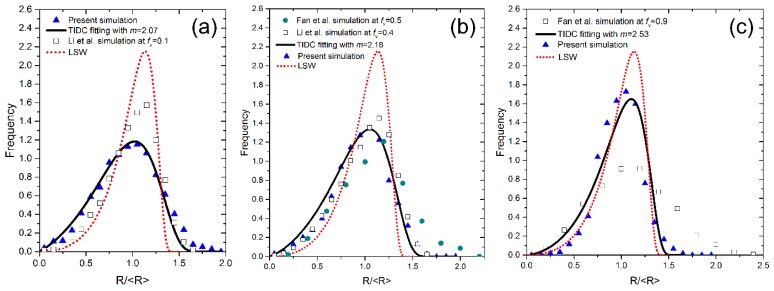
Comparison of the (near) steady state PSDs of the present results with the simulation results by Li et al. [12], Fan et al. [10] and the prediction of LSW theory at the volume fraction of (**a**) 10%; (**b**) 50%; (**c**) 90%. The solid lines are the theoretical PSDs of the TIDC theory with different m values that best fit with the present simulation results. PSD: particle size distribution; LSW: Lifshitz, Slyozov and Wagner; TIDC: trans-interface-diffusion-controlled.

As aforementioned, the steady state of Ostwald ripening is hardly reached for a single simulation. To obtain the PSDs in the steady state as far as possible, the initial microstructures with more particles (about 6000) for the volume fractions of 10%, 50% and 90% were re-generated according to the PSDs of last simulations, and calculations with these newly initial microstructures were carried out again until the particles number decreases to about 1500. Now the PSDs are much closer to the steady state and can be compared to the relevant theory predictions, as shown in Figure 7. Apparently, the theoretical PSD of the LSW theory is a much poorer fit to the present results, while the theoretical PSDs of the TIDC theory with the exponent m of 2.07, 2.18 and 2.53 fit the present results quite well at the volume fraction of 10%, 50% and 90%, respectively. The present results are quite different from the phase field simulation results of Li et al. [18] and Fan et al. [16], since these phase field models actually describe the matrix diffusion limited case, which is fundamentally different from the current CA model that describes the interface-transfer limited case.

### 3.2. Rayleigh Instability

**Figure 8:**
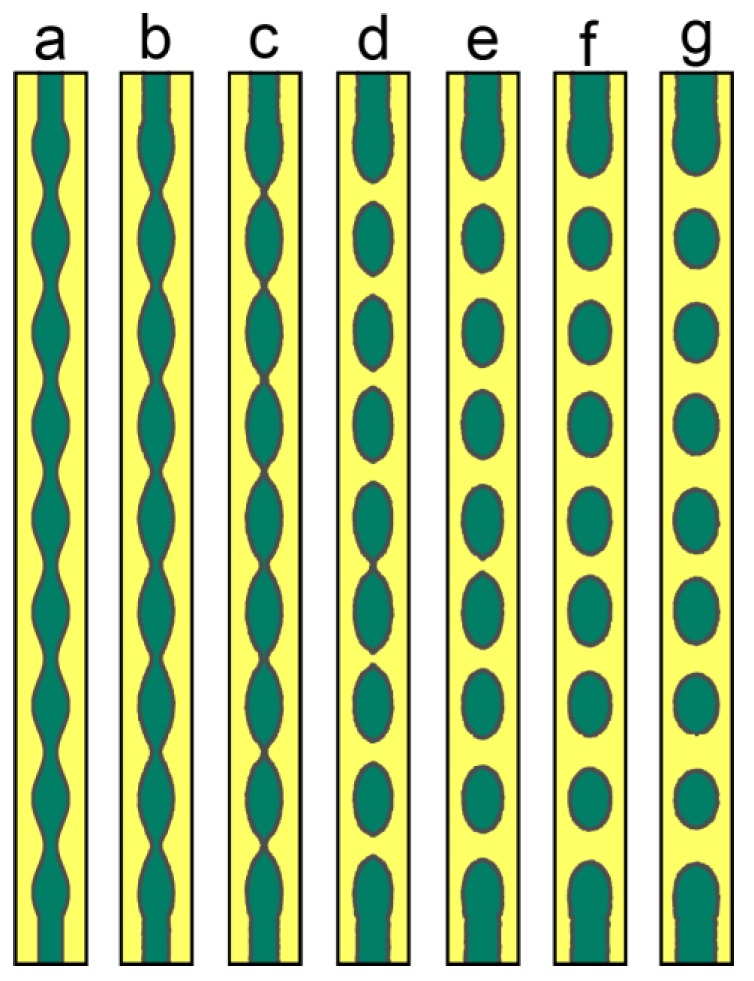
Microstructure evolution of a long cylinder with sinusoidal perturbation on the surface. (**a**) *t* = 0 s; (**b**) *t* = 0.4 s; (**c**) *t* = 0.7 s; (**d**) *t* = 1 s; (**e**) *t* = 1.2 s; (**f**) *t* = 1.4 s; (**g**) *t* = 1.6 s. Simulation grid is 3000 × 240 with the cell size of 0.001 μm.

The most studied case of Rayleigh instability is a long cylinder with perturbation on the surface. Figure 8 shows the microstructure evolution of such a long cylinder with initial sinusoidal perturbation on the surface. Due to the curvature difference at the convex region and the concave region, there will be surface flux from the concave region to the convex region and the interface contracts at the concave region, and eventually breaks into separated particles. The classical theory of Rayleigh instability predicts a critical wavelength of *λ* > 2π*R*_0_, beyond which the Rayleigh instability is possible. Recently, Wang and Nestler [29] demonstrated that critical wavelength should be $\lambda =2\pi \sqrt{R_{0}^{2}-\delta^{2}}$, where *δ* is the amplitude of the perturbation, which is more generalized, and the modified theory was confirmed by their phase field simulations. In the current work, we do not mean to examine the critical condition of Rayleigh instability, and we just set perturbation with large enough wavelength, making sure the breakup of the cylinder can occur.

**Figure 9:**
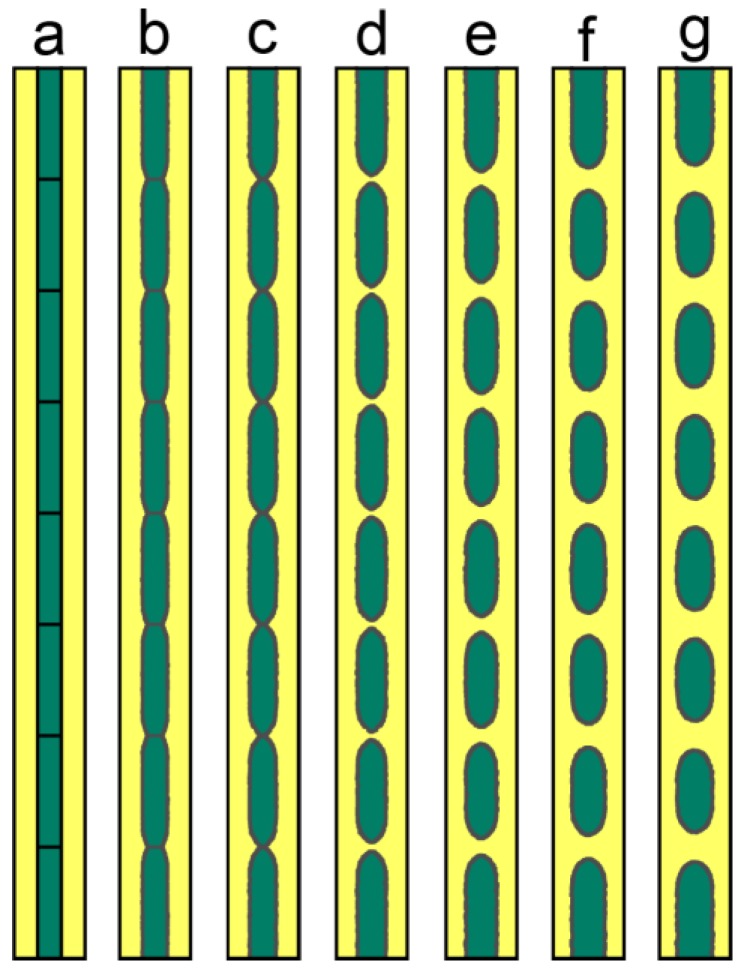
Microstructure evolution of a long cylinder with inner substructures. (**a**) *t* = 0 s; (**b**) *t* = 0.2 s; (**c**) *t* = 0.4 s; (**d**) *t* = 0.6 s; (**e**) *t* = 0.8 s; (**f**) *t* = 1 s; (**g**) *t* = 1.2 s. Simulation grid is 3000 × 240 with the cell size of 0.001 μm.

Long cylinder phase of crystalline material may contain grain boundaries within it, and this case is also considered. Figure 9 shows the microstructure evolution of a long cylinder with inner grain boundaries. The presence of grain boundary in the cylinder phase causes grain grooving, and the groove deepens with time, and finally ruptures the cylinder phase. Although the two cases are apparently different, both of them lead to similar broken of the cylinder, since both perturbation on the surface, and inner grain boundaries, can change the geometry and energetics of the system and can evolve driven by the reduction of interface energy.

**Figure 10:**
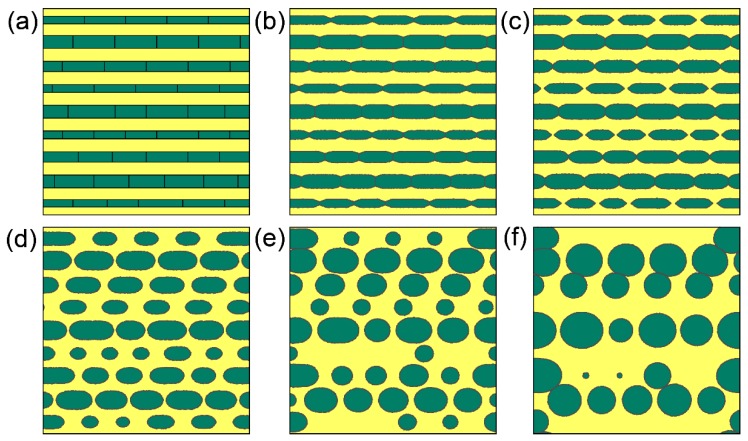
Microstructure evolution of a system with long cylinders with inner substructures, which shows a combination of Rayleigh instability and Ostwald ripening. (**a**) *t* = 0 s; (**b**) *t* = 0.2 s; (**c**) *t* = 0.4 s; (**d**) *t* = 1 s; (**e**) *t* = 2 s; (**f**) *t* = 4 s. Simulation grid is 1600 × 1600 with cell size of 0.001 μm.

In reality, a system may contain many long cylinders, and each cylinder may have different perturbations at the interface or have different inner substructures. Figure 10 shows the microstructure evolution of a system with long cylinders with inner grain boundaries. Purposely, various thicknesses of cylinders with different arrangements of inner grain boundaries are set in one system. Grain boundaries grooving occurs first and breaks the cylinders into low-aspect-ratio cylinders. Thin cylinders are less stable than thick cylinders and are broken up ahead of thick cylinders. After the breakup, interfaces become much rounder, and small particles shrink and disappear, and large particles become larger, which is the case of Ostwald ripening. So Rayleigh instability and Ostwald ripening successively occur in a system with long cylinders, and the whole process is referred to as spheroidization. Structures containing high-aspect-ratio features, e.g., lamellar structure, tend to spherize at elevated temperatures, which is a common phenomenon in many engineering materials. The present CA model is so general that it is suitable to be applied to materials with the microstructure evolution related to Ostwald ripening and Rayleigh instability as long as the kinetic mechanism of the material is consistent with the model. More specifically, it is a promising model to be used in the simulation of the static spheroidization process of two-phase titanium alloys with a lamellar structure at elevated temperature. As CA method can be conveniently coupled with crystal plasticity FEM simulations of the hot-working process of titanium alloys [64], the model can also be extended to simulate the dynamic spheroidization of titanium alloys with a lamellar structure during hot deformation.

## 4. Conclusions

A cellular automata model for modeling both Ostwald ripening and Rayleigh instability in two-phase systems based on the curvature-driven mechanism has been developed in this paper. Varying temporal growth exponent m in the range of 2 and 3 is predicted for transient Ostwald ripening. The coarsening rate constant shows independence on the volume fraction of coarsening phase. PSDs evolve during transient Ostwald ripening, and wide PSD becomes narrowed toward steady state, while narrow PSD becomes widened at first and then narrowed. The simulated results of Ostwald ripening are more consistent with the TIDC theory rather the LSW theory, since the presented CA model actually describes the interface-transfer limited case. Shrinkage of a single particle with kinetic exponent m close to 2 also demonstrates that the present CA model captures the interface-transfer mechanism. Rayleigh instabilities of cylinders with sinusoidal perturbation on the surface and with inner grain boundaries are also successfully simulated with the same CA model. Successively occurrence of Rayleigh instability and Ostwald ripening in a system containing long cylinders with interior grain boundaries was also simulated, showing a capability of the current CA model to simulate the spheroidization process of complex microstructure like lamellar structure.

## Figures and Tables

**Table 1:** Key parameters used in the simulations [42,48,58].

Parameter	Value	Unit
*M*_0_	0.5	mmol∙J^−1^·s^−1^
*Q_αγ_*	147	kJ∙mol^−1^
*Q*_b_	120	kJ∙mol^−1^
*γ*_m_	0.56	J·m^2^
*χ*	110	J∙(at.%)^−1^∙mol^−1^
*V*_m_	7.09 × 10^−6^	m^3^∙mol^−1^
*x*_0_	0.4	wt.%
*b*	2.58 × 10^−10^	m
